# Identification, High-Density Mapping, and Characterization of New Major Powdery Mildew Resistance Loci From the Emmer Wheat Landrace GZ1

**DOI:** 10.3389/fpls.2022.897697

**Published:** 2022-05-13

**Authors:** Zuzana Korchanová, Miroslav Švec, Eva Janáková, Adam Lampar, Maciej Majka, Kateřina Holušová, Georgi Bonchev, Jakub Juračka, Petr Cápal, Miroslav Valárik

**Affiliations:** ^1^Centre of the Region Haná for Biotechnological and Agricultural Research, Institute of Experimental Botany of the Czech Academy of Sciences, Olomouc, Czechia; ^2^Department of Cell Biology and Genetics, Faculty of Science, Palacký University Olomouc, Olomouc, Czechia; ^3^Faculty of Natural Sciences, Comenius University in Bratislava, Bratislava, Slovakia; ^4^Institute of Plant Genetics, Polish Academy of Sciences, Poznań, Poland; ^5^Institute of Plant Physiology and Genetics, Bulgarian Academy of Sciences, Sofia, Bulgaria; ^6^Department of Computer Science, Faculty of Science, Palacký University Olomouc, Olomouc, Czechia

**Keywords:** wheat, powdery mildew (*Blumeria graminis* D. C. f. sp. *tritici*), resistance, emmer, GZ1, QTL mapping

## Abstract

Powdery mildew is one of the most devastating diseases of wheat which significantly decreases yield and quality. Identification of new sources of resistance and their implementation in breeding programs is the most effective way of disease control. Two major powdery mildew resistance loci conferring resistance to all races in seedling and adult plant stages were identified in the emmer wheat landrace GZ1. Their positions, effects, and transferability were verified using two linkage maps (1,510 codominant SNP markers) constructed from two mapping populations (276 lines in total) based on the resistant GZ1 line. The dominant resistance locus *QPm.GZ1-7A* was located in a 90 cM interval of chromosome 7AL and explains up to 20% of the trait variation. The recessive locus *QPm.GZ1-2A*, which provides total resistance, explains up to 40% of the trait variation and was located in the distal part of chromosome 2AL. The locus was saturated with 14 PCR-based markers and delimited to a 0.99 cM region which corresponds to 4.3 Mb of the cv. Zavitan reference genome and comprises 55 predicted genes with no apparent candidate for the *QPm.GZ1-2A* resistance gene. No recessive resistance gene or allele was located at the locus before, suggesting the presence of a new powdery mildew resistance gene in the GZ1. The mapping data and markers could be used for the implementation of the locus in breeding. Moreover, they are an ideal base for cloning and study of host–pathogen interaction pathways determined by the resistance genes.

## Introduction

Hexaploid bread wheat (*Triticum aestivum* subsp. *aestivum*, 2n = 6× = 42, AABBDD) and tetraploid durum (pasta) wheat (*Triticum turgidum* subsp. *durum*, 2n = 4× = 28, AABB) are significant commercial grain crops worldwide. Their high and stable yield is the most important aspect of food security. Nevertheless, wheat yields could be threatened by various fungal diseases such as powdery mildew, rust, or Fusarium head blight (Chrpová et al., 2013; Bansal et al., 2020; Dreiseitl, 2021). Powdery mildew, caused by *Blumeria graminis* (DC.) E.O. Speer f. sp. *tritici* is one of the most devastating fungal diseases which decreases the yield and quality of susceptible varieties by up to 40% (Dreiseitl, 2011). Growing resistant cultivars is an effective way to control powdery mildew infections, as it represents the most economical and environmentally safe approach to eliminate the use of fungicides. Unfortunately, intensive breeding narrowed down the bread wheat gene pool (Feuillet et al., 2008). Therefore, the introduction of resistance genes derived from wheat landraces and related species into agriculturally used cultivars is an attractive way of wheat gene pool enrichment.

To date, more than 70 *Pm* genes and their alleles were identified in 60 loci across different species of wheat (Li et al., 2019; McIntosh et al., 2019) and most of them are major resistance genes (R-genes). In most cases, the R-genes code protein receptors with leucine-rich repeats (LRR), which recognize proteins secreted by the pathogen. They are usually dominant and short-living following the gene-for-gene concept (Flor, 1971). On the other hand, more durable resistance that is effective against a broader range of races, but often incomplete (called slow mildewing), could be provided by the involvement of a single locus with several resistance genes (Janáková et al., 2019) or by multiple minor resistance genes (Van Der Plank, 1963; Kou and Wang, 2010). The coding of this type of resistance is often dispersed into multiple discrete quantitative trait loci (QTLs). Nevertheless, loci with long-lasting resistance resembling the non-host resistance and effective against all tested isolates were observed. One of them is the *Lr34* (*Yr18/Pm38*) locus carrying a gene with pleiotropic effect (ABC transporter) conferring resistance to various fungal pathogens, such as powdery mildew, leaf rust, and stripe rust (Krattinger et al., 2009). Another one, the *Mlo* locus identified in barley, is effective against all races throughout the season and encodes a plant membrane protein (Büschges et al., 1997). The *Mlo* resistance is determined as homozygous-recessive and has been successfully employed in agriculture for more than 40 years. So far, only one such gene has been identified and is used exclusively in spring cultivars to minimize the probability of its breakage.

However, the use of the R-genes in plant breeding forces strong selection pressure on pathogen populations. Therefore, this type of resistance is generally short-term and, in the case of cultivated crops, tends to be effective for about 3–5 years before it loses efficiency (Wolfe and McDermott, 1994; Dreiseitl, 2003). Moreover, the frequently observed quantitative nature of non-specific resistance makes it difficult to handle in breeding programs (Kou and Wang, 2010). The main reason is the problematic co-inheritance of all minor genes after crossbreeding, which can result in lower efficiency of the resistance. The scarcity of durable and race-independent resistance genes requires a continuous search for new sources of resistance to powdery mildew. Recently, a total resistance against a wide range of powdery mildew races at all growth stages was identified in tetraploid emmer wheat landrace GZ1 (*Triticum turgidum* subsp. *dicoccum*). Here, we present the mapping and characterization of the major loci involved in the GZ1 resistance against powdery mildew.

## Materials and Methods

### Plant Material

The powdery mildew-resistant spring emmer wheat line GZ1 (*T. turgidum* subsp. *dicoccum*) was collected during the expedition in Sobotište na Myjave in Slovakia and provided by Ing. Štefan Masár (Research Institute of Plant Production, Piešt’any, Slovakia). The dicoccum lines Eichenbarlebener (winter, EBL) and DIM25 (spring, PI 94633) were obtained from the Plant Breeding and Acclimatization Institute (IHAR)—National Research Institute (Radzików, Poland) and the United States Department of Agriculture (Beltsville, MD, United States), respectively. F_1_ hybrids were created from a cross of the GZ1 line with the EBL and DIM25 lines, where GZ1 was used as pollen donor (to eliminate possible effects of GZ1 cytoplasm). F_2_ mapping populations were derived from progenies of the F_1_ hybrids.

### Powdery Mildew Resistance Assessment

Leaf segments of the lines GZ1, EBL, and DIM25 were inoculated individually with 30 isolates derived from the powdery mildew population captured by stationary collection at Bratislava, Slovakia, in May 2004. From 2005 to 2020, the GZ1, EBL, and DIM25 lines were sown annually in the autumn in field conditions at Bratislava-Prievoz, Slovakia.

Isolates A17, A24, and A3ab of *Blumeria graminis* (DC) E.O. Speer f. sp. *tritici* (Bgt) were used for phenotyping of the parental lines and mapping populations. The isolates were selected based on their aggressiveness (the shortest time from spore inoculation of plant material to the formation of conidiophores). All isolates originate from a collection of Bgt at the Research Institute of Plant Production in Piešt’any. In the preliminary testing on the parental and F_1_ lines (data not shown), all isolates performed without significant difference and indicated a single homozygous-recessive gene conferring broad-spectrum resistance. To diminish the influence of increased phenotype variability due to single isolate fitness and virulence genes composition, a mixture (1:1:1) of all three isolates was used for phenotyping.

Mapping reliability was enhanced by phenotyping F_2:3_ progeny according to which the original phenotype of each F_2_ line was reconstructed as a codominant marker. About 20 seedlings of each F_2:3_ line were grown in plastic pots filled with peat. After 10 days, the primary leaves were cut into 2.5 cm long segments and deposited on Petri dishes filled with 0.5% agar medium containing 862 mg/L of benzimidazole (Sigma-Aldrich, St. Louis, MO, United States). The inoculated leaf segments were incubated for 13 days in a growth chamber under continuous light (800 lux) at 18°C. Subsequently, the response to powdery mildew was visually evaluated as the presence of colonies on all F_2:3_ lines (susceptibility, score 0), the absence of colonies on all segments (resistance, score 1), or colonies only on several lines (heterozygous, score 0.5). Deviation from Mendelian inheritance was evaluated using the chi-square goodness of fit test (Pearson, 1900).^1^

### Genotyping and Linkage Map Construction

DNA was extracted from dried (37°C for at least 24 h) 2 cm long young leaf segments. The dry leaf segments were homogenized in the presence of two glass balls (5 mm) at 27 Hz for 3 min using a MM301 mill (Retsch, Haan, Germany). DNA was extracted and purified using a NucleoSpin^®^ Plant II kit (Macherey-Nagel, Düren, Germany) and quantified with a NanoDrop 2000 (Thermo Fisher Scientific, Waltham, MA, United States). DNA was genotyped using the DArTseq approach at Diversity Arrays Technology Pty., Ltd. (Canberra, Australia)^2^ and the obtained sequencing data were analyzed together for both mapping populations. Markers were subjected to quality testing followed by the removal of markers with more than 10 missing data points or significant segregation distortion (over 30%). Only codominant polymorphic DArTseq markers were used for map construction.

Linkage maps were constructed using MultiPoint UltraDense v4.1 (MultiQTL Ltd., Haifa, Israel^3^; Ronin et al., 2017). DArTseq single nucleotide polymorphism (SNP) markers were preliminarily filtered and processed as an F_2_ population with default settings. Markers with more than four missing data points and χ^2^-values greater than 10 (*p* = 0.05) were removed. Markers were clustered into multiple linkage groups (LGs) of ordered co-segregating markers using a guided evolutionary strategy (GES) algorithm (Ronin et al., 2010) with 10 jackknife re-sampling runs. After the first clustering, the 14 longest LGs were selected for subsequent analysis due to the tetraploid character of *T. turgidum*. Markers that disrupted the monotony and caused unstable neighborhoods were checked for segregation ratios, linkage distances, missing data, and associations with other markers, and were eventually removed to stabilize the LGs until the global variation value decreased below 1.1. Subsequently, the LGs were saturated with additional markers from the Heap group using the “Extending linkage group” function with the coefficient of enlargement increased stepwise from 1.0 to 1.2. The order of markers was rechecked for monotony distortion and map size enlargement, and those causing disruption were eliminated. Only the “skeleton” maps were used for subsequent analyses. The resulting LGs were exported to Microsoft Excel with recombination frequencies converted to centiMorgans (cM) using the Kosambi mapping function (Kosambi, 1943).

Individual linkage groups were assigned to a particular chromosome based on the wheat DArTseq consensus map (Diversity Arrays Technology Pty., Ltd., Canberra, Australia, see text footnote 2). Exported LGs were visualized in MapChart v2.32 (Voorrips, 2002).

### Quantitative Trait Loci Analysis

Mapping of quantitative trait loci (QTLs) was performed independently for both mapping populations using MultiQTL v2.6 (MultiQTL Ltd., Haifa, Israel, see text footnote 3, Korol et al., 2001). The skeleton maps, which contain only the most informative skeleton markers, were used. QTL mapping was carried out by multiple interval mapping (MIM, Kao et al., 1999) using single-QTL per chromosome sub-models. QTL LOD threshold values, standard deviations, and 95% confidence intervals (CI) of QTL positions were estimated with bootstrap analysis (10,000 iterations). QTLs were declared significant when their LOD scores exceeded the respective *p* < 0.01 critical LOD thresholds. QTL effects were determined as the percentage of explained variance (PEV) of the trait relative to its phenotypic variation.

### Marker Development, Candidate Gene Identification, and 2A Map Saturation

The 2A chromosomes from all three parental lines were flow-sorted from liquid suspensions of mitotic chromosomes prepared from the root tips of seedlings as described by Vrána et al. (2000). The chromosome samples were fluorescently labeled by FISHIS using (GAA)_7_ oligonucleotides labeled by Alexa488 on both termini (Integrated DNA Technologies, Coralville, IA, United States) and counterstained by DAPI (4′,6-diamidino-2-phenylindole) as described by Giorgi et al. (2013). Bivariate flow karyotyping and chromosome sorting were performed on a FACSAria II SORP flow cytometer and sorter (Becton Dickinson Immunocytometry Systems, San Jose, CA, United States). Contamination of sorted fractions by other chromosomes was determined using fluorescence *in situ* hybridization (FISH) using probes for telomeric repeats, Afa repeat, and (GAA)n, according to Kubaláková et al. (2003). Before DNA sequencing, 100,000 flow-sorted chromosomes were treated with 60 ng/ml proteinase K (Roche, Basel, Switzerland) for 40 h at 50°C, and DNA was purified using a Microcon YM-100 column (MilliporeSigma, Burlington, MA, United States) as described by Šimková et al. (2008). About 20 ng of purified DNA was fragmented in 20 μl using a Bioruptor Plus (Diagenode, Liège, Belgium) five times for 30 s at the HIGH setting. The sequencing libraries were prepared from sheared DNA using a NEBNext Ultra II DNA Library Prep Kit for Illumina (New England Biolabs, Ipswich, MA, United States) with the following modification: (i) size selection was directed for a larger final library size (∼1,000 bp), (ii) PCR enrichment was done in nine cycles, (iii) library was size-selected using a BluePippin (Sage Science, Beverly, MA, United States) in pre-cast 1.5% agarose gel cassettes. The library was sequenced on a NovaSeq 6000 (Illumina, San Diego, CA, United States) and 2 × 250 bp paired-end reads were produced. The raw data were trimmed for low-quality bases using Trimmomatic (Bolger et al., 2014) and assembled to scaffolds with Meraculous v2.0.5 (Chapman et al., 2011) using 111-bp k-mers. Scaffolds shorter than 1 kb were discarded.

Candidate genes for GZ1 powdery mildew resistance loci were identified by anchoring the mapped QTLs to the cv. Zavitan reference genome sequence (Avni et al., 2017). Sequences of markers from identified QTLs were BLASTN (Altschul et al., 1990) aligned using default parameters. Only the best blast hits were considered. Annotated genes from regions delimited by the alignments were extracted from a list of high-confidence annotated genes (Avni et al., 2017).

PCR markers for map saturation were designed according to Janáková et al. (2019) with minor modifications. Sequences of selected genes were BLASTN aligned with the assemblies of all three chromosomes 2A and identified scaffolds were compared. Only low-copy sequences with nucleotide identity greater than 95% were considered for marker development, and those containing short indels were preferred. If no indel was identified, markers were designed based on SNPs. PCR primers flanking the polymorphism were designed using Primer3web v4.1.0 (Untergasser et al., 2012). The *Xgwm294* marker was used for genotyping according to Mohler et al. (2013). All primer pairs were tested on all parental lines.

The PCR reaction mix (15 μl) contained 0.01% (*w/v*) *o*-cresolsulphonephtalein, 1.5% (*w/v*) sucrose, 0.2 mM of each dNTP, 0.6 U of Taq DNA polymerase, 1 μM of each primer, 10 mM Tris-HCl, 50 mM KCl, 1.5 mM MgCl_2_, 0.1% (*v/v*) Triton X-100, and 10–20 ng of genomic DNA. The reaction conditions consisted of an initial denaturation (95°C/5 min), followed by 40 cycles of 95°C/30 s, an optimized annealing temperature (Table 1) for 30 s, and 72°C for 30 s. The PCR reactions were completed with an elongation step of 72°C/10 min. The amplicons were electrophoretically separated on 4% non-denaturing polyacrylamide gels using a Mega-Gel Apparatus C-DASG-400-50 (C.B.S. SCIENTIFIC, CA, United States) and were stained with ethidium bromide and visualized with an InGenius LHR2 Gel Imaging System (Synoptics, Cambridge, United Kingdom).

**TABLE 1:** Markers used for the *QPm.GZ1-2A* locus saturation.

Marker	Forward primer (5′→3′)	Reverse primer (5′→3′)	Ta (°C)	Type^b^
*owm2005*	TGACTCATGGCATGGCACACGT	AAAAGTCATTACCATCAACTG	60	CAPS (*Mlu*I, *Bst*UI)
*owm2016* ^a^	CACATCCGATTTGTCGTCAG	CCACTAATTCTGCAAGTACACTCC	60	CAPS (*Bsm*I)
*owm2023*	GACCTCTCGACGGTGGATAA	TTTTAAAAATCATGCAAGAAATAAGAG	60	CAPS (*Hae*III)
*owm2026*	CCCTGAAGGGAGGGAACTT	ATCGAAGGGACCCTCTGTCT	60	CAPS (*Bsm*AI)
*owm2027*	ACATTGGTGAAGCATGCAGA	TGAGGCTGTCAGATGGTGTC	60	CAPS (*Alu*I)
*owm2028*	AAGAGGTAGCACACGGATGG	CATGCATGCGTTTGTTCG	60	CAPS (*Hha*I)
*owm2033*	CGTCTGCTGGATTTAGCATT	GAGCAAACATGCCGAACAC	60	CAPS (*Nla*III)
*owm2034*	ATCTGATGGAGTCCGTGGG	TGGCCGGCTAAAGAAAGTTG	60	CAPS (*Rsa*I)
*owm2037* ^a^	CAGCCTCCATTGTTGCAAGA	ACGGGGACTATGGCTTGTAG	60	CAPS (*Alu*I)
*owm2063*	CATGAGTCGACAGTGCGTG	TCTGGGTGAGTAGATTTCTGC	60	INDEL (4 bp)
*owm2066*	TGCCGCACATTTTATAGACACC	ACCTCCAATGGCATCGAGAA	60	CAPS (*Sac*II)
*owm2067*	TGGAGATGCAGTGGGACTTT	TGAAGGGTTGGGAGTGGAAA	60	CAPS (*Rsa*I)
*owm2077*	GGATGAAGAAGGCGCTCAAG	GAATGACAGCGTGTGGGATC	60	INDEL (14 bp)
*owm2079*	CACATGGAAGGGAACACTGC	GCACATTTCAGTTTGCCACC	60	CAPS (*Bst*NI)

*^a^Markers owm2016 and owm2037 were derived from the original QTL flanking DArTseq markers 996555-38 and 2276132-44, respectively.*

*^b^Restriction enzymes used for the detection of polymorphisms are in the brackets.*

## Results

### GZ1, DIM25, and EBL Reaction to Powdery Mildew

After screening with the 30 isolates, only GZ1 showed complete resistance to all isolates used in the screening test. The EBL and DIM25 showed susceptibility to all isolates. Annual sowing of the three lines at the locality Bratislava, Slovakia, resulted in frequent infections of the EBL and DIM25 lines. In all 15 seasons, on the GZ1 line, no symptoms of the powdery mildew were observed.

### Mapping Populations and Powdery Mildew Resistance Analysis

Two F_2_ mapping populations were derived from crosses of the resistant GZ1 line with two susceptible cultivars, EBL and DIM25, comprising 125 and 151 F_2_ lines, respectively. These two populations were used for QTL mapping and verification, and also, testing of the trait’s transferability to different genetic backgrounds. To increase the reliability of phenotyping for mapping purposes and enhance the survival of genotyped F_2_ lines, about 20 seedlings from subsequent F_2:3_ families were phenotyped and F_2_ phenotypes were deduced as codominant markers.

The GZ1 × EBL F_2_ population comprised 16 susceptible, 34 resistant, and 63 heterozygous individuals. In the remaining 12 lines, significant contamination with *Aspergillus* fungi was observed and were considered unreliable. According to χ^2^ of 7.09 (*p* = 0.0289), the segregation ratio does not fit the expected Mendelian segregation ratio for a single gene inheritance (1:2:1). The verification GZ1 × DIM25 F_2_ population comprised 15 susceptible, 59 resistant, and 73 heterozygous lines. Four lines altered by *Aspergillus* contamination were ignored; χ^2^ was 26.35 (*p* = 0.0000019) confirming the distortion from the 1:2:1 ratio; therefore, QTL analysis was performed with these phenotype data.

**FIGURE 1:**
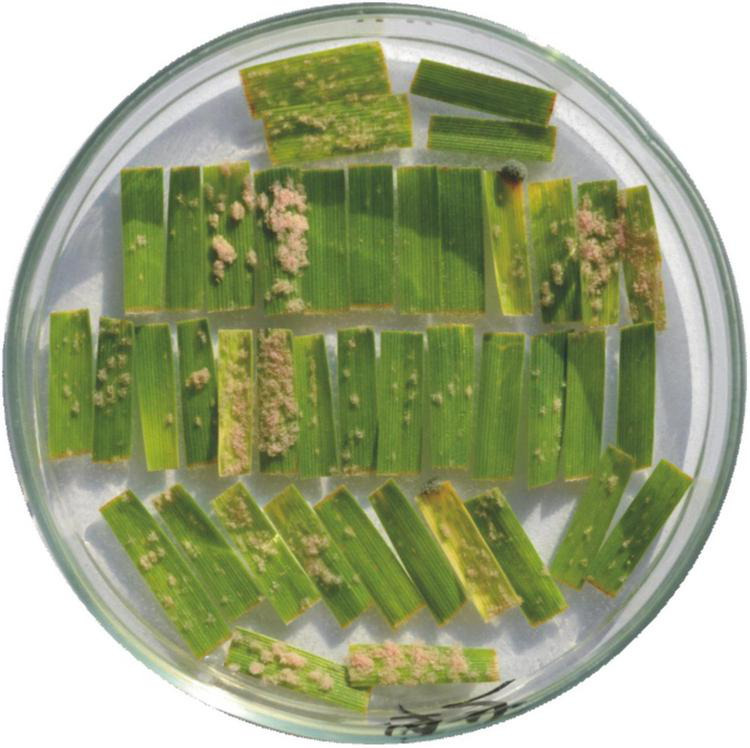
Observed reaction to powdery mildew infection. The first line (horizontal) represents two segments of susceptible line EBL (left) and two segments of resistant line GZ1 (right). The remaining leaf segments (vertical) represent tested F_2:3_ families, always two primary leaf segments of a different plant of the same family (counted from the left upper corner). Plant reaction to powdery mildew: resistant (no colonies, e.g., segments 5–8, and 25–26) or susceptible (with colonies). Among the susceptible plants, there is a difference in the reaction. Some segments were completely infected and covered with large colonies (e.g., segments 3 and 4), while on others, there were only small colonies (e.g., segments 1 and 2).

In contrast to preliminary phenotyping, the F_2:3_ phenotype analysis revealed two types of susceptibility reactions. Besides resistance reaction with no colonies, small and large colonies were also observed (Figure 1).

### Genetic Map Construction

A total of 23,012 SNP markers were obtained from the DArTseq analysis for both mapping populations. Data filtering was done individually for each mapping population. After quality and normal segregation (χ^2^) filtering, a set of 7,985 and 6,218 SNP markers (2,291 shared markers) was used for the construction of GZ1 × EBL and GZ1 × DIM25 linkage maps.

The skeleton map of GZ1 × EBL comprises 14 linkage groups with 862 skeleton markers (Supplementary Table 1) and a genetic length of 3,102.54 cM. An average marker density is 3.69 cM. The length of the linkage groups ranged from 186.99 cM (chromosome 4B) to 262.11 cM (chromosome 7A) with an average of 221.61 cM. The individual linkage groups comprised 62 skeleton markers on average, with the highest number of markers (88) on chromosome 7A and the lowest (38) on chromosome 4B. The lowest and highest marker densities were observed for chromosomes 4B (4.92 cM) and 2B (2.64 cM), respectively (Table 2).

**TABLE 2:** GZ1 × EBL and GZ1 × DIM25 skeleton linkage maps.

	GZ1 × EBL			GZ1 × DIM25		
Chromosome	Length (cM)	Marker density (cM/marker)	Number of markers	Length (cM)	Marker density (cM/marker)	Number of markers
1A	195.52	3.76	52	94.17	3.92	24
2A	220.58	3.80	58	168.77	3.31	51
3A	242.33	4.33	56	193.14	4.20	46
4A	188.82	3.56	53	113.68	3.44	33
5A	251.52	4.12	61	175.24	4.74	37
6A	202.18	4.04	50	113.31	3.24	35
7A	262.11	2.98	88	216.57	3.38	64
1B	192.85	3.21	60	192.54	3.21	60
2B	192.50	2.64	73	188.73	4.19	45
3B	255.68	3.50	73	222.11	3.70	60
4B	186.99	4.92	38	81.12	3.86	21
5B	237.50	4.09	58	216.25	3.49	62
6B	243.12	3.24	75	220.12	4.40	50
7B	230.84	3.45	67	182.11	3.04	60
A genome	1563.06	3.80	418	1074.88	3.75	290
B genome	1539.48	3.58	444	1302.98	3.70	358
Total	3102.54	−	862	2377.86	−	648
Average	221.61	3.69	62	169.85	3.72	46

GZ1 × DIM25 skeleton map was constructed using 648 markers (Supplementary Table 2) and spanned 2,377.86 cM with an average marker density of 3.72 cM. The length of the linkage groups ranged from 81.12 cM (chromosome 4B) to 222.11 cM (chromosome 3B) with an average of 169.85 cM. The linkage groups comprised 46 markers on average. Similarly, to the previous map, the highest number of markers (64) was assigned to chromosome 7A, while chromosome 4B contained the lowest number of markers (21). Chromosomes 5A and 7B had the lowest (4.74 cM) and the highest (3.04 cM) marker density, respectively (Table 2).

#### Quantitative Trait Loci Analysis

Quantitative trait loci mapping revealed four genomic regions associated with the resistance on chromosomes 2A, 3A, 3B, and 7A. QTLs located on chromosomes 3A and 3B had low LOD scores and were not detected in both mapping populations. Therefore, they were not verified and were abandoned. However, QTLs located on chromosomes 2A (denominated *QPm.GZ1-2A*) and 7A (denominated *QPm.GZ1-7A*) were significant in both mapping populations. *QPm.GZ1-2A* had a LOD score of 15.65 (GZ1 × EBL) and 20.8 (GZ1 × DIM25) and explained 31.7 and 39.6% of phenotypic variance (PEV) for this trait, respectively. *QPm.GZ1-7A* reached the LOD score of 9.7 (GZ1 × EBL, PEV 20.9%) and 7.3 (GZ1 × DIM25, PEV 11%; Table 3).

**TABLE 3:** QTLs related to *Pm* resistance in the GZ1 × EBL and GZ1 × DIM25 mapping populations.

Population	QTL	Chr	LOD	LOD threshold	*p*-value	QTL peak position (cM)	Confidence interval (95%)	PEV^a^ (%)
**GZ1 × EBL**	*QPm.GZ1-2A*	2A	15.65 ± 3.29	3.66	0.0005	159.65 ± 4.10	151.6–167.7	31.7 ± 4.8
	*QPm.GZ1-7A*	7A	9.7 ± 2.57	3.72	0.0005	248.12 ± 14.48	219.7–262.0	20.9 ± 4.5
**GZ1 × DIM25**	*QPm.GZ1-2A*	2A	20.8 ± 3.89	3.42	0.00008	155.44 ± 0.77	153.9–156.9	39.6 ± 4.9
	*QPm.GZ1-7A*	7A	7.29 ± 2.59	3.55	0.00008	204.25 ± 40.02	125.8–216.6	11.0 ± 3.6

*^a^PEV, percentage of explained variance.*

**FIGURE 2:**
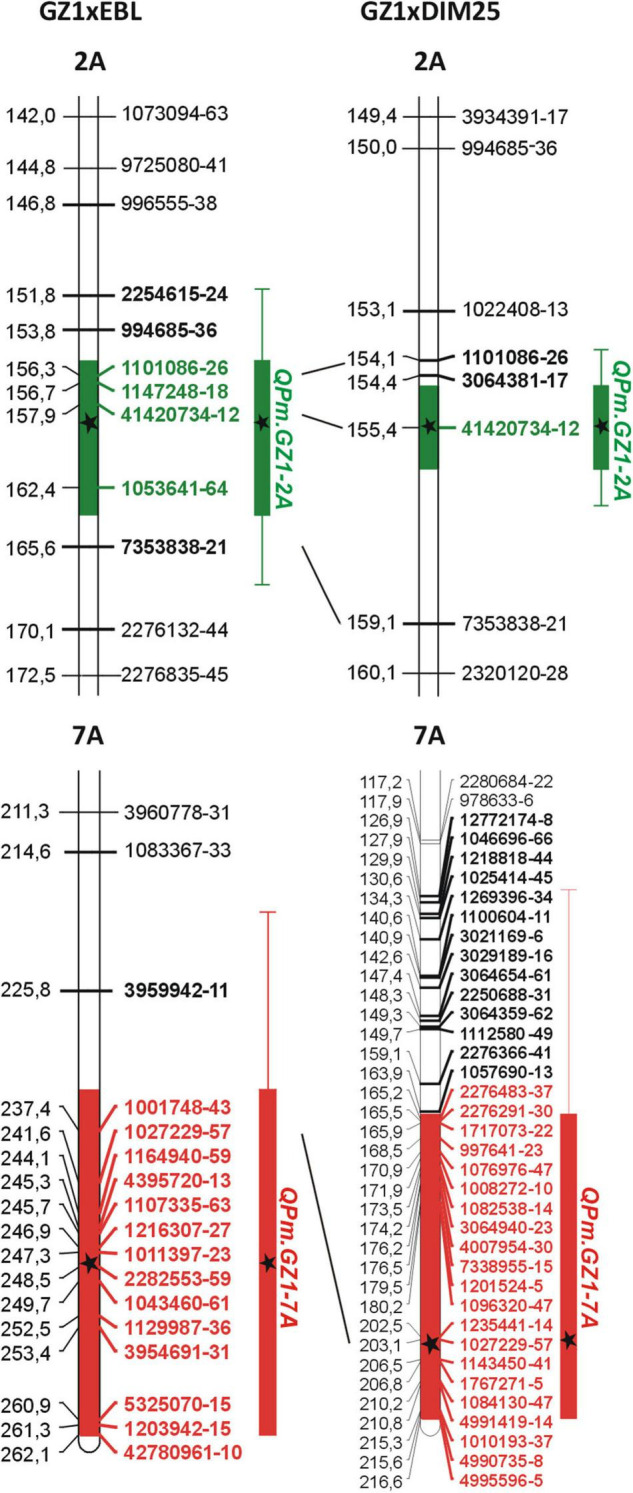
Highly significant QTLs for powdery mildew resistance mapped in the GZ1 × EBL and GZ1 × DIM25 mapping populations. Only relevant parts of chromosomes with mapped QTLs are shown. The genetic distances (left side) are in cM (Kosambi). In the **GZ1 × EBL**, the ***QPm.GZ1-2A*** was in the region from 155.55 to 163.75 cM (8.2 cM; green bar) of chromosome 2AL with the QTL peak at 159.65 cM (black star). This region comprises 4 markers (bold green). The confidence interval extended the area from 151.6 to 167.7 cM (16.1 cM; thin green line). This 16.1 cM long region comprises 7 markers (bold). In the **GZ1 × DIM25**, ***QPm.GZ1-2A*** was in a 1.54 cM long region (154.67–156.21 cM; green bar) with one marker (bold green). The QTL peak is at 155.44 cM (black star) of chromosome 2AL. The confidence interval extended the area of this QTL to 3 cM (153.9–156.9 cM; thin green line and 3 markers in bold). In the **GZ1 × EBL**, ***QPm.GZ1-7A*** was in a 28.96 cM long region (red bar) of the chromosome 7AL and comprises 14 markers (bold red). Confidence interval extends the area to 42.3 cM (thin red line) with 15 markers (bold). The QTL peak is at 248.12 cM (black star). In the **GZ1 × DIM25**, the ***QPm.GZ1-7A*** was in a 52.34 cM long region (red bar, 21 markers) of the chromosome 7AL, with the confidence interval of 90.8 cM (red thin line) comprising 35 markers (bold) with the QTL peak at 204.25 cM (black star). The shared markers are indicated by lines connecting the maps.

The QTL analysis of the GZ1 × EBL population placed the *QPm.GZ1-2A* locus in the 151.6–167.7 cM interval of chromosome 2A (16.1 cM) with a peak at 159.65 cM (Figure 2 and Table 3). The region comprises 7 markers and is flanked by markers *996555-38* and *2276132-44* (Supplementary Table 1 and Figure 2). The syntenic region in the reference genome of cv. Zavitan (Avni et al., 2017) delimited by the flanking markers (Supplementary Table 3 and Figure 2) covers 22.7 Mb (696,152,174–718,833,310 bp) on chromosome 2AL. In the GZ1 × DIM25 verification mapping population, the *QPm.GZ1-2A* locus was placed in a 153.9–156.9 cM region with a peak at 155.44 cM (Figure 2 and Table 3). It comprises 3 markers and is flanked by markers *1022408-13* and *7353838-21* (Supplementary Table 2 and Figure 2). This region corresponds to 10.9 Mb (700,992,011–711,927,608 bp) of the cv. Zavitan chromosome 2A and completely overlaps with the GZ1 × EBL locus (Supplementary Table 4 and Figure 2).

The *QPm.GZ1-7A* in the GZ1 × EBL map was located on chromosome 7AL within a 42.3 cM interval (219.7–262.0 cM) with a peak at 248.12 cM (Figure 2 and Table 3). The region comprises 15 markers and is flanked by markers *1083367-33* and *42780961-10* (Supplementary Table 1 and Figure 2). The syntenic locus in the cv. Zavitan genome delimited by the flanking markers covers 47 Mb (678,080,238–725,069,898 bp; Supplementary Table 5). In the GZ1 × DIM25 map, the *QPm.GZ1-7A* was in a 90.8 cM interval (125.8–216.6 cM) with a peak at 204.25 cM and is flanked by markers *12772174-8* and *4995596-5* (Supplementary Table 2 and Figure 2). The region comprises 35 markers (Figure 2 and Supplementary Table 6) and corresponds to 143.8 Mb (564,777,524–708,600,178 bp) of the cv. Zavitan chromosome 7AL and overlays the GZ1 × EBL *QPm.GZ1-7A* locus (Supplementary Tables 5, 6).

#### *QPm.GZ1-2A* and *QPm.GZ1-7A* Interactions

**FIGURE 3:**
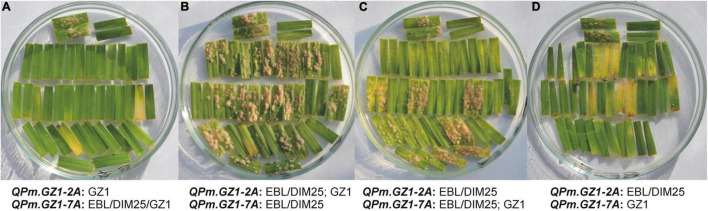
Phenotypic reaction of F_2:3_ families to a mixture of powdery mildew isolates A17, A23, and A3ab focusing on the interaction between *QPm.GZ1-2A* and *QPm.GZ1-7A*. Examples of phenotypic reactions of individual F_2_ lines with allelic states of the resistance loci as indicated under the pictures (GZ1 = homozygous and resistant, EBL/DIM25/GZ1 = any allele, EBL/DIM25 = homozygous and susceptible, EBL/DIM25; GZ1 = heterozygous). In all pictures, the top two pairs of horizontally placed segments are susceptible control EBL (left) and resistant control GZ1 (right). For all F_2_ lines, about 20 F_2:3_ progenies were tested and each of them is represented by two leaf segments. **(A)** F_2_ lines homozygous for the GZ1 allele at the *QPm.GZ1-2A* irrespective of the *QPm.GZ1-7A* showed complete resistance. **(B)** Lines that were heterozygous at *QPm.GZ1-2A* and carry the homozygous susceptible allele at *QPm.GZ1-7A* segregated in a ratio of 1 (resistant): 3 (susceptible). **(C)** Lines heterozygous at *QPm.GZ1-7A* and homozygous susceptible at *QPm.GZ1-2A* segregated in a ratio of approximately 3 (resistant): 1 (susceptible). Of 21 F_2:3_ progeny lines, leaf segments of seven plants (7, 9, 12, 15, 18, 20, 21) were covered with large colonies, while segments of eight plants (1, 5, 6, 10, 11, 13, 17, 19) had very small colonies The remaining six plants (2, 3, 4, 8, 14, 16) had almost no visible colonies. **(D)** Lines that were homozygous for the GZ1 allele at the *QPm.GZ1-7A* but homozygous for the susceptible parent at the *QPm.GZ1-2A* inhibited infection of powdery mildew almost completely.

Interactions between these two QTLs were examined by comparing phenotypic data of lines with different genomic compositions at the resistance loci. The homozygous-recessive constitution of the GZ1 genotype at the *QPm.GZ1-2A* locus was in all cases associated with total resistance to powdery mildew with no respect to genotype constitution at *QPm.GZ1-7A* (Figure 3A). Progeny testing of individual F_2_ lines confirmed that *QPm.GZ1-2A* is recessively inherited because the heterozygous constitution at the *QPm.GZ1-2A* locus with homozygous EBL or DIM25 genotype at *QPm.GZ1-7A* resulted in plants with large colonies or without colonies (Figure 3B) to the advantage of susceptible ones. The homozygous constitution (EBL or DIM25) at the *QPm.GZ1-2A* locus with the heterozygous constitution at the *QPm.GZ1-7A* locus resulted in segregation for large colonies, small colonies, and no colonies to the advantage of resistant ones, suggesting that the *QPm.GZ1-7A* is a dominant gene (Figure 3C). The homozygous constitution of EBL or DIM25 at the *QPm.GZ1-2A* locus with homozygous GZ1 constitution at *QPm.GZ1-7A* was associated with the presence of a few small colonies or no colonies (Figure 3D), suggesting that the phenotype is mediated by dosage-dependent gene effects.

#### Map Saturation at the *QPm.GZ1-2A* Resistance Locus

The major resistance locus *QPm.GZ1-2A* was selected for saturation (Supplementary Tables 2, 3). As stated above, the *QPm.GZ1-2A* locus was delimited to the corresponding 22.7 Mb region of the cv. Zavitan reference genome sequence, which comprises 316 predicted genes (Supplementary Table 3). The locus was saturated with 14 new markers and the marker *Xgwm294* associated with the *Pm50* gene (Supplementary Table 7). Marker development was facilitated by sequencing the 2A chromosomes of the parental lines. The assemblies of the GZ1, DIM25, and EBL chromosomes 2A comprise 365, 366, and 364 Mb with N50 of 26.1, 24.3, and 23.3 kb, respectively. The saturation allowed to delimit the *QPm.GZ1-2A* resistance locus to a region between markers *owm2016* and *1101086-26*. This region is 0.99 cM long and corresponds to 4.3 Mb of the cv. Zavitan genome (Supplementary Table 7) with 55 annotated (Supplementary Table 3) high-confidence genes (excluding transposon related genes). Of these, three genes have a known relation to resistance (Supplementary Table 3).

## Discussion

Tetraploid wheat *Triticum turgidum* subsp. *dicoccum* GZ1 was studied for its broad-range race non-specific resistance to *Blumeria graminis* (DC) E.O. Speer f. sp. *tritici*. Analysis of F_1_ hybrids indicated that the powdery mildew resistance might be controlled by a single recessive gene due to the susceptible reaction of all individuals (data not shown). Race non-specific recessive resistance is very rare because most of the resistance genes are usually coded by dominant R-genes following the gene-for-gene concept (Flor, 1971). Major R-genes provide resistance to only one or a few races (race-specific resistance). Race non-specific resistance, effective against a wide range of races, is usually determined by several genes (Van Der Plank, 1963). However, in some cases, race non-specific resistance could also be controlled by major genes, as it is in the case of barley *Mildew resistance locus o* (*Mlo*). The loss of function of both alleles of a single *Mlo* gene in barley was found to confer recessively inherited broad-spectrum resistance at all growth stages (Jørgensen, 1992). A similar reaction to powdery mildew was observed due to a recessive mutation of a single gene in the tetraploid GZ1-based hybrids (Figure 1). However, in hexaploid bread wheat, all *Mlo*-like genes in all three homoeologous genomes must be silenced to achieve resistance, as was shown by Wang et al. (2014) using knockout of the gene family by TALENs. This is a strong difference from the GZ1 gene. Additionally, the *Mlo* resistance is characterized by cell wall appositions, papillae formation at the encounter sites, early chlorophyll decay, and spontaneous mesophyll cell death leading to chlorosis and leaf necrosis negatively affecting yield (Bayles et al., 1990; Peterhansel et al., 1997; Piffanelli et al., 2002; Makepeace et al., 2007). In the case of the GZ1 resistance, leaf necrosis was not observed supporting the hypothesis of different resistance mechanisms, which makes the gene highly attractive for mapping and cloning.

The susceptible winter and spring cvs. EBL and DIM25, respectively, were used for the construction of mapping populations and confirmed transferability of the GZ1 resistance to different genetic backgrounds. In contrast to original assumptions of the presence of a single resistance gene, a reproducible shift from 1:2:1 segregation ratio was observed. Moreover, employing the F_2:3_ lines allowed the identification of three reaction types (Figure 1). Both observations surprisingly indicated the presence of more than one resistance gene. The accumulation of several resistance genes in a single line is quite common and the accumulation of several major resistance genes is highly desirable or even created by breeders using the gene pyramiding approach (Koller et al., 2018).

The DArTseq genotyping of the GZ1-derived F_2_ mapping populations provided sufficient marker density for subsequent QTL analysis (Table 2). Both maps have size and marker density (Table 2) comparable with published tetraploid wheat maps (e.g., Peleg et al., 2008). Due to an unexpected deviation of phenotypic data from the Mendelian segregation ratio, what suggests polygenic inheritance of the trait, the QTL analysis was applied. The QTL analysis identified two highly significant loci contributing to the resistance variation. Their LOD scores significantly exceeded their respective LOD threshold values (Table 3). Powdery mildew resistance of GZ1 was found to be controlled by the *QPm.GZ1-2A* and *QPm.GZ1-7A* loci on the chromosome 2AL and 7AL, respectively. Reliability of mapping of both resistance loci was confirmed by their detection in both populations in overlapping positions (Figure 2 and Supplementary Tables 3, 4). This high-quality mapping could be attributed to categorical phenotype assessment of the F_2_ lines using the F_2:3_ progenies. Even though the conversion of phenotype to a codominant marker provides reliable mapping which eliminates the residual variability (environmental influences), it is suitable only for mapping of a limited number of loci with a large contribution to phenotype variance. This approach cannot match the resolution achieved by scaling (e.g., Dreiseitl, 2022); therefore, polygenic resistance determined by a higher number of loci with a small contribution to phenotype may not be detected and verified due to the influence of the residual variability.

In this study, a homozygous-recessive resistance conferred by a single gene was expected, so the less sensitive but more robust phenotyping approach was employed. The suitability of this phenotyping approach was confirmed by reliable and verified mapping of two major resistance loci as stated above. Each mapped locus showed distinct phenotypic manifestation. Homozygous-recessive *QPm.GZ1-2A* confers total resistance with no respect to *QPm.GZ1-7A* (Figure 3A). The progeny of plants dominant for the 2A and heterozygous for the 7A locus shows segments with large colonies, small colonies, or no visible colonies (Figure 3C). In the case of the homozygous-dominant constitution of loci on chromosomes 2A and 7A, symptoms manifest as small colonies or no visible colonies, suggesting an additive effect of the alleles at the 7A locus (Figure 3D). However, further work is required to verify this effect.

**FIGURE 4:**
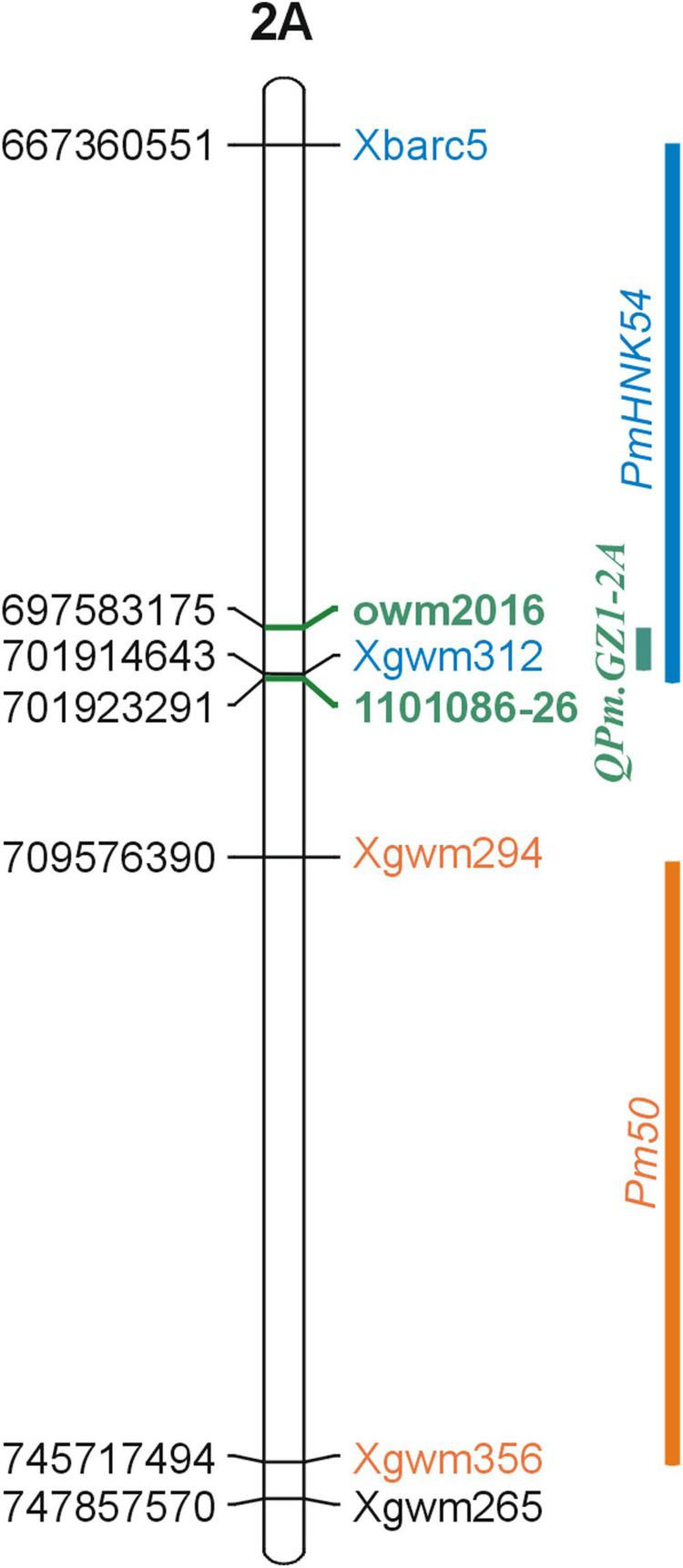
Genomic comparison of known powdery mildew resistance loci adjacent to the *QPm.GZ1-2A* locus. The reference genome sequence of cv. Zavitan was used to analyze the *QPm.GZ1-2A* locus position on the genome level. The *QPm.GZ1-2A* locus (green bar) is flanked by the *owm2016* and *1101086-26* markers (green) overlapping the *PmHNK54* locus (Xu et al., 2011, blue) and neighboring the *Pm50* locus. Mohler et al. (2013) located the *Pm50* 3.8 cM distally from the *Xgwm294* marker and it does not overlap with the *QPm.GZ1-2A* locus. *PmHNK54* was mapped 6 cM proximally to the *Xgwm312* marker. The *QPm.GZ1-2A* and *PmHNK54* loci share about 4.3 Mb of the 31 Mb *PmHNK54* region between the *Xgwm312* and *Xbarc5* markers; however, *PmHNK54* was found to be dominant and is different from the recessive *QPm.GZ1-2A* locus.

To date, eight powdery mildew resistance genes have been identified on chromosome 2AL. These include formally named multi-allelic *Pm4* (Briggle, 1966), and tentatively designated genes for powdery mildew such as *PmLK906* (Niu et al., 2008), *PmHNK54* (Xu et al., 2011), *PmPS5A* (Zhu et al., 2005), *PmYm66* (Hu, 2008), *Pm50* (Mohler et al., 2013), *PmHo* (Komáromi et al., 2016), and *pmX* (Fu et al., 2013). *Pm4* consists of four resistance alleles *Pm4a*, *Pm4b*, *Pm4c*, and *Pm4d* (The et al., 1979; Hao et al., 2008; Schmolke et al., 2011). The homology of the *QPm.GZ1-2A* locus with loci of all these genes was determined by examining their genomic positions according to their co-segregating markers. The direct comparison revealed that only *PmHNK54*, which was located together with *QPm.GZ1-2A* between the *Xbarc5* and *Xgwm312* markers maps to the same region (Figure 4). However, this gene was found to be dominant (Xu et al., 2011), whereas GZ1 resistance was so far characterized as recessive. Therefore, *QPm.GZ1-2A* is distinguishable from *PmHNK54*.

The *QPm.GZ1-7A* was mapped to a large region of chromosome 7AL (144 Mb). So far, about 20 *Pm* genes have been localized on chromosome 7AL: *Pm1* consisting of five different alleles: *Pm1a*, *Pm1b*, *Pm1c* (*Pm18*), *Pm1d* (Hsam et al., 1998), and *Pm1e* (*Pm22*; Singrün et al., 2003), *Pm9* (Schneider et al., 1991), *mlRd30* (Singrün et al., 2004), *Pm37* (Srnić et al., 2005; Perugini et al., 2007), *NC96BGTA4* (Srnić et al., 2005), *MlIW172* (Ouyang et al., 2014), *PmU* (Qiu et al., 2005), *NCA6* (Miranda et al., 2007), *Mlm2033* and *Mlm80* (Yao et al., 2006; Liang et al., 2016), *MlIw72* (Ji et al., 2007), *MlAG12* (Maxwell et al., 2009), *MlWE18* (Han et al., 2009), *PmG16* (Ben-David et al., 2010), *PmTb7A.1* and *PmTb7A.2* (Chhuneja et al., 2011), *HSM1* (Li et al., 2013), *MlUM15* (Worthington et al., 2014), *Pm59* (Tan et al., 2018), and *QPm.gb-7A* (Desiderio et al., 2021). According to Desiderio et al. (2021), most of these genes are likely to be closely linked and create clusters. Most known R-genes encode immune receptors from the nucleotide-binding site leucine-rich repeats (NBS-LRR) protein family. Resistance genes are abundant in plant genomes and clustering was observed (International Wheat Genome Sequencing Consortium (IWGSC), 2018). *QPm.GZ1-7A* also resides in such a resistance gene-enriched region. However, this could be influenced by the large confidence interval of *QPm.GZ1-7A*, and therefore, additional work is necessary to differentiate between the already designated powdery mildew resistance genes and the *QPm.GZ1-7A* locus. Almost all genes, except *Pm9* (Schneider et al., 1991) and *mlRD30* (Singrün et al., 2004), are major dominant (R-genes). The physical interval retrieved for *QPm.GZ1-7A* in cv. Zavitan (46.9 Mb, Supplementary Table 5) comprises 34 disease resistance-related genes including nine from the LRR family. As mentioned above, *QPm.GZ1-7A* is dominant, suggesting that it could be an NBS-LRR-like gene.

The recessive character of *QPm.GZ1-2A* and its strong resistance effect make it a more attractive source of resistance compared to the *QPm.GZ1-7A*, and therefore, only the *QPm.GZ1-2A* was selected for further map saturation. The flanking markers of the GZ1 × EBL region (Supplementary Tables 1, 3) were used as the starting points. The 22.7 Mb long *QPm.GZ1-2A* region of the GZ1 × EBL map was saturated with 14 new markers (Table 1). The final *QPm.GZ1-2A* region (about 4.3 Mb) in the cv. Zavitan reference genome sequence (Avni et al., 2017) is flanked by the *owm2016* and *1101086-26* markers (Supplementary Table 7). The narrowed down *QPm.GZ1-2A* region does not overlap with the QTL peaks (associated with the 41420734*-12* marker, Figure 2) predicted by the QTL analysis. The observed proximal shift (Supplementary Table 7) could be attributed to noise in the phenotype data caused by the presence of two resistance genes.

The *QPm.GZ1-2A* region comprises 55 annotated genes (Supplementary Table 3) and none of them have any relation to the *Mlo* gene family. Additionally, the *Mlo* gene was mapped on chromosome 4H (Simons et al., 1997) and is not orthologous to the *QPm.GZ1-2A* mapped on chromosome 2A supporting the previous assumptions that *QPm.GZ1-2A* is different from the *Mlo* gene. However, three of these genes have relation to the resistance genes involved in pathogen attack signaling. Since genes from the signaling pathways are mostly dominant R-genes (e.g., Peart et al., 2005; Sánchez-Martín et al., 2016), there is only a small probability that the recessive *QPm.GZ1-2A* is one of them. However, one of them could be the *PmHNK54* gene (Xu et al., 2011). Nevertheless, confirmation of the assumptions would require further work.

The broad-range resistance in all stages of plant development mediated by the single major *QPm.GZ1-2A* locus makes it extremely attractive to breeders and the high-density genetic map of the locus offers molecular markers for its effective implementation in breeding programs. Moreover, the results provide an ideal base for cloning and study of the novel recessive gene determining the resistance.

## Data Availability Statement

The datasets presented in this study can be found in online repositories. The names of the repository/repositories and accession number(s) can be found in the article/Supplementary Material.

## Author Contributions

MV, ZK, and MŠ designed the study. ZK, AL, and MM were responsible for marker development, genotyping, map construction, and data analysis. MŠ and EJ were responsible for the construction of mapping populations. MŠ, GB, and ZK performed the phenotypic evaluation. AL performed the statistical analysis. PC sorted the chromosomes and extracted chromosome-specific DNA. KH was responsible for survey sequencing and sequence assembly. ZK, MV, and JJ conducted the bioinformatics analyses. ZK conducted the other experiments. ZK and MV drafted the manuscript. All authors contributed to its editing and proofreading.

## Conflict of Interest

The authors declare that the research was conducted in the absence of any commercial or financial relationships that could be construed as a potential conflict of interest.

## Publisher’s Note

All claims expressed in this article are solely those of the authors and do not necessarily represent those of their affiliated organizations, or those of the publisher, the editors and the reviewers. Any product that may be evaluated in this article, or claim that may be made by its manufacturer, is not guaranteed or endorsed by the publisher.

## Footnotes

1
graphpad.com/quickcalcs/chisquared1.cfm

2
diversityarrays.com

3
https://www.graphpad.com/quickcalcs/chisquared1.cfm
